# Health care providers’ ethical perspectives on waiver of final consent for Medical Assistance in Dying (MAiD): a qualitative study

**DOI:** 10.1186/s12910-022-00745-4

**Published:** 2022-01-30

**Authors:** Caroline Variath, Elizabeth Peter, Lisa Cranley, Dianne Godkin

**Affiliations:** 1 Lawrence S. Bloomberg Faculty of Nursing, University of Toronto, 155 College Street, Suite 130, Toronto, ON M5T 1P8 Canada; 2 Joint Centre for Bioethics, University of Toronto, 155 College Street, Suite 754, Toronto, ON M5T 1P8 Canada; 3 Faculty of Health Sciences and Wellness, Humber College ITAL, 205 Humber College Blvd, Toronto, ON M9W 5L7 Canada; 4 Regional Ethics Program, Trillium Health Partners - Mississauga Hospital, 100 Queensway W, Mississauga, ON L5B 1B8 Canada

**Keywords:** Medical Assistance in Dying, Consent, Decision-making capacity, Healthcare providers, Relational autonomy, Moral agency, End-of-life care

## Abstract

**Background:**

With the enactment of Bill C-7 in Canada in March 2021, people who are eligible for medical assistance in dying (MAiD), whose death is reasonably foreseeable and are at risk of losing decision-making capacity, may enter into a written agreement with their healthcare provider to waive the final consent requirement at the time of provision. This study explored healthcare providers’ perspectives on honouring eligible patients’ request for MAiD in the absence of a contemporaneous consent following their loss of decision-making capacity.

**Method:**

A critical qualitative methodology, using a feminist ethics theoretical lens with its focus on power and relationality, was used to examine how socio-political and environmental contexts influenced healthcare providers' moral agency and perspectives. Semi-structured interviews were conducted with 30 healthcare providers (13 physicians, six nurse practitioners, nine nurses and two social workers) from across Canada who provide MAiD-related care.

**Results:**

Themes identified include; (1) balancing personal values and professional responsibilities, (2) anticipating strengths and limitations of the proposed waiver of final consent amendment, (3) experiencing ethical influences on decisions to enter into written agreements with eligible patients, (4) recognizing barriers to the enactment of MAiD in the absence of a contemporaneous consent and (5) navigating the potential for increased risks and burden.

**Discussion:**

To our knowledge, this is the first study in Canada to explore healthcare providers’ perspectives on waiving the final consent for MAiD using a written agreement. Most participants supported expanding eligible people’s access to MAiD following loss of capacity, as they believed it would improve the patients’ comfort and minimize suffering. However, the lack of patients’ input at the time of provision and related ethical and legal challenges may impact healthcare providers’ moral agency and reduce some patients’ access to MAiD. Providers indicated they would enter into written agreements to waive final consent for MAiD on a case-by-case basis. This study highlights the importance of organizational, legal and professional support, adequate resources, clear policies and guidelines for the safety and wellbeing of healthcare providers and to ensure equitable access to MAiD.

## Background

### Canadian legislation on medical assistance in dying

In 2016, the Canadian government passed Bill C-14, which introduced changes to the Criminal Code to allow for the provision of medical assistance in dying (MAiD) [1, 2]. Eligibility requirements for MAiD under Bill C-14, included: (1) being eligible for funded healthcare services in Canada; (2) being 18 years of age or older; (3) having a grievous and irremediable medical condition including a reasonably foreseeable natural death; (4) making a voluntary request; and (5) being able to provide informed consent (Table 1) [2]. A safeguard included in Bill C-14 was the requirement to confirm consent immediately prior to MAiD provision [2]. A ten-day (minimum) reflective wait-period between patients’ written request and the scheduled date of MAiD provision was required under this law; this wait-period could only be shortened if patients were at imminent risk of death or loss of capacity as determined by two assessors [2].

**Table 1:** Bill C-14 eligibility criteria and safeguards (previous legislation)

BILL C-14 eligibility criteria
18 + years of age
Eligible for publicly funded health services
Has decision-making capacity at the time of request and to provide final consent
Informed consent to receive MAID given after patient informed of means available to relieve suffering
Voluntary request for MAID
Person has “grievous and irremediable medical condition”, meaning:
serious and incurable illness, disease or disability and has an advanced state of irreversible decline in capability;
has enduring physical or psychological suffering that is intolerable to them and cannot be relieved under conditions that they consider acceptable
Their natural death has become reasonably foreseeable

Bill C-14 safeguards
Patient must make a written request that must be witnessed and signed by two independent witnesses
Witness may not be a family member or a health care worker
Two independent practitioners must confirm all eligibility criteria
Person must be informed that they can withdraw request at any time, by any means
Mandatory 10-day reflection period after written request is signed, unless death or loss of capacity imminent
Immediately before MAID is provided, person must be given opportunity to withdraw consent
Must confirm consent at the time of provision to receive MAID

Government of Canada [6]

The law indicates that only physicians and nurse practitioners may assess a person’s eligibility for MAiD and provide MAiD either directly through administration of intravenous medications or through prescription of an oral drug that the individual self-administers [1, 2]. Others such as registered nurses, social workers, spiritual care practitioners can assist and provide technical skills and support [3, 4]. Some organizations have introduced a new role of MAiD Coordinator. Their role is to coordinate, educate and support the MAiD request and provision process for assessors, providers, patients, and their families [3, 4].

In 2020, the Canadian Government tabled further changes to the Criminal Code through Bill C-7 which came into effect in March 2021. The main change introduced with Bill C-7 is that it enables persons whose natural death is not reasonably foreseeable to access MAiD, if they meet all other eligibility criteria [5–7]. Bill C-7 introduced two tracks with differing procedural safeguards for persons requesting MAiD based on whether the patient’s natural death was reasonably foreseeable or not (Table 2) [5]. Track-1 safeguards are used for persons who meet all eligibility requirements whose natural death is reasonably foreseeable [5]. Track-2 safeguards, which include a 90-day assessment period and the person’s express consent at the time of MAiD provision, are required for persons whose natural death is not reasonably foreseeable. These changes were in response to support from the public as identified through a nationwide survey, consultation with stakeholders from across the nation and the outcome of the Superior Court of Quebec’s decision on the case presented by plaintiffs Truchon and Gladu to remove the reasonably foreseeable natural death criterion [5, 6]. In that case, the plaintiffs who experienced intolerable suffering from progressive neurological conditions, successfully argued that the requirement in Bill C-14 that natural death be “reasonably foreseeable” violated their Charter rights to access MAiD, as they were unable to meet this criterion [5].

**Table 2:** Bill C-7 eligibility criteria and safeguards (current legislation)

BILL C-7 eligibility criteria
18 + years of age
Eligible for publicly funded health services
Has decision-making capacity
Informed consent to receive MAID given after patient informed of means available to relieve suffering
Voluntary request for MAID
Person has “grievous and irremediable medical condition”, meaning:
serious and incurable illness, disease or disability and has an advanced state of irreversible decline in capability;
has enduring physical or psychological suffering that is intolerable to them and cannot be relieved under conditions that they consider acceptable
Person whose natural death is reasonably foreseeable (track-1 safeguards) Person whose natural death is not reasonably foreseeable (track-2 safeguards)

BILL C-7 TRACK-1 SAFEGUARDS (For persons whose death is reasonably foreseeable)	BILL C-7 TRACK-2 SAFEGUARDS (For persons whose death is not reasonably foreseeable)
Patient must make a written request that must be witnessed and signed by one independent witness Two independent practitioners must confirm all eligibility criteria Person must be informed that they can withdraw request at any time, by any means No reflection period Immediately before MAID is provided, person must be given opportunity to withdraw consent Ensure that the person gives express consent to receive medical assistance in dying **Final express consent confirmation can be waived if consent agreement is given in advance**^a^	Patient must make a written request that must be witnessed and signed by one independent witness Two independent practitioners must confirm all eligibility criteria Ensure that the assessors consult with a medical practitioner or nurse practitioner who has that expertise in the condition the person is suffering from, and share the results of that consultation with the two assessors Ensure that the healthcare provider have discussed with the person the reasonable and available means to relieve the person’s suffering Ensure that there are at least 90 clear days between the day eligibility criteria is established and the MAiD provision date Person must be informed that they can Withdraw request at any time, by any means Immediately before MAID is provided, person must be given opportunity to withdraw consent Ensure that the person gives express consent to receive medical assistance in dying

^a^Focus of this study

Government of Canada [6] and Parliament of Canada [7]

### Waiver of final consent (using a written agreement)

Another key change introduced with Bill C-7, and the focus of this study, is that persons whose death is reasonably foreseeable who are at risk of losing capacity may agree to waive the final consent requirement of Bill (C-14) (Table 2) [5, 7]. The waiver of final consent can occur only if the person’s natural death is reasonably foreseeable and they: (1) have met all eligibility requirements; (2) have been informed that they are at risk of losing capacity; (3) have identified the date that they wish to receive MAID, and (4) have entered into a written agreement to waive final consent with the providing medical or nurse practitioner should they lose capacity prior to the date of provision; and (5) do not resist the administration of lethal medications at the time of provision [5–7]. The ten-day reflective wait-period was removed for persons whose natural death is reasonably foreseeable [7].

### Advance requests for MAiD

In Canada, the changes in the Criminal Code through Bill C-7 do not extend to advance requests for MAiD for persons who are not otherwise currently eligible or have already lost capacity, and therefore differs from legislation in other jurisdictions (Table 3). Belgium, Luxemburg, The Netherlands, and Columbia allow for the provision of MAiD using advance requests made by patients prior to meeting eligibility criteria, to be used when patients suffer from a serious and incurable disorder and have lost decision-making capacity [1]. There is minimal evidence on the experiences with the implementation of advance requests in these jurisdictions [1, 8]. Some have identified challenges with the use of advance requests, specifically with establishing the criteria of suffering following the loss of capacity and patients’ refusal of MAiD [9–13]. There is less controversy in Belgium and Luxemburg as MAiD can only be provided using an advance request to those who are in an irreversible state of unconsciousness [1]. Furthermore, there is evidence that the rate of honouring advance requests and the number of reported deaths using advance requests for MAiD is low in some of these jurisdictions [1, 13–15]. In Canada a survey indicates that 62–74% of the general public support the availability of advance requests for MAiD [1]. Experts in Canada highlight the need for research on end-of-life practices and decision-making to consider the expansion of access using advance requests [1].

**Table 3:** Comparison of legal criteria for the use of advance consents/waiver of final consent

Criteria	Countries				
	Belgium	Colombia	Luxembourg	The Netherlands	Canada
Created after meeting all eligibility requirements					x
Must be in writing	x	x	x	x	x
Must be written by the person requesting MAiD	x	x	x	x	x
Must be witnessed	x		x		x
Must be discussed with a physician/Nurse Practitioner					x
Must be registered in a national registry	x		x		
Patient’s case must be assessed by independent healthcare providers	x	x	x	x	x
Must be discussed with the patient’s SDMs	x	x	x		
Is valid for five years after they are signed	x		x		
Should set a mutually agreed upon date					x
May only be followed for irreversibly unconscious patients	x		x		
Should not be honoured if patient demonstrates meaningful resistance					x
Must be evaluated by an oversight committee		x			
Deaths must be reported to an oversight committee	x	x	x	x	

Adapted from CCA [1]

Previous studies on healthcare providers’ perspectives on and willingness to provide MAiD using advance requests have focused almost exclusively on patients with dementia. These studies reveal that most healthcare providers expressed concern about the provision of MAiD using advance requests from patients with dementia [16–20]. In Canada eligible patients at risk of experiencing loss of capacity secondary to conditions including, but not limited to, dementia, cancer, neurodegenerative disorders and stroke, can enter into a written agreement to waive final consent for MAiD only if they currently meet all other eligibility criteria. Canadian healthcare providers’ perspectives on and willingness to provide MAiD to eligible patients in the absence of a contemporaneous final consent, as proposed in the Bill C-7 have not been explored.

The purpose of this study was to explore: (1) Canadian healthcare providers’ perspectives on providing MAiD to eligible patients in the absence of a contemporaneous final consent following their loss of decision-making capacity; and (2) how power and relationality shaped their moral agency with respect to entering into and honouring a written agreement to waive final consent for MAiD.

## Study design

### Methodology

A critical approach using concepts from feminist ethics was chosen to guide this study.

Prevailing power relations and the social contexts and vulnerabilities of healthcare providers and those with loss of decision making capacity in relation to accessing MAiD in the absence of a contemporaneous consent were examined [21–23]. Critical research is guided by key ontological underpinnings such as: (1) assumptions and ideologies inform all research; (2) overt, subtle, and covert dimensions of power exist within society; (3) reality is made up of contradictions rather than consensus; and 4) experiences are dialectic and are influenced by socio-political and cultural contexts [21].

Feminist ethics problematizes concepts such as health, healing, and medicine from the premise that social relations are unequal and hierarchical [24]. Using a feminist ethics lens to focus on the concepts of power, relationality, and moral agency provided a deeper understanding of the healthcare providers’ perspectives examined in this study [25]. Power imbalances are enhanced when patients lose decision-making capacity [23]. Healthcare providers are also subject to power dynamics, depending on the socio-political context they work in, and their relationships with others involved in patients’ care [25]. Applying a feminist ethics lens also helped us to understand their perspectives by focusing on relational influences such as relationships and socio-political contexts on their experiences, behaviours, and decisions [23]. Exploring participants’ moral agency, i.e., their capacity to recognize, deliberate/reflect on, and act on moral responsibilities” (p. 221) [26], provided further insight into their perspectives, influences on decisions to participate in MAiD, and the risk for moral distress [27].

Substantive appraisal guidelines that highlight the importance of reflexivity and the creative use of theoretical foundations of the study offered by Eakin and colleagues were used to uphold rigour [28]. Substantive reviewers acknowledge the role of the researchers’ subjectivity in creating and influencing the research process [28–30]. The first author (CV) is a registered nurse who was part of a MAiD team at the onset of this study. The second author (EP) is a nurse ethicist who has drawn on feminist ethics extensively in her scholarship. The third author (LC) focuses her research on the decision-making of residents in long-term care. The fourth author (DG) is an ethicist who developed and directs a MAiD team for a large regional institution. Collectively, all the authors have extensive experience in end-of-life research.

### Participants

A maximum variation approach of purposive sampling method was used to capture core experiences from the various settings in which MAiD is practiced across Canada, using established inclusion criteria (Table 4) [31]. Invitations to participate were sent via email listservs and flyers posted on social media platforms of professional and voluntary organizations. In addition, snowball sampling methods were used to maximize heterogeneity [31]. Data was collected from 30 healthcare providers in Canada (Table 5). All participants had experiences with patients who had met the eligibility requirements for MAiD but had lost decision-making capacity before MAiD could be provided. The sample-size enabled an in-depth analysis of divergent experiences of a similar phenomenon from across Canada and is consistent with substantive appraisal guidelines [28, 31].

**Table 4:** Participant inclusion criteria

	Inclusion criteria	Exclusion criteria
Types of Participants	Healthcare providers in Canada who were directly involved in the care of eligible patients who were unable to receive MAiD due to their loss of decision-making capacity Healthcare providers may include physicians, nurse practitioners, nurses, social workers, spiritual care practitioners, ethicists, speech language pathologists, etc English speaking healthcare providers	Healthcare providers employed at Trillium Health Partners Lay-caregivers

**Table 5:** Demographic data

Participant (*n* = 30) characteristics	
Professional role	
Nurse Practitioners	6
Physicians	13
Registered Nurses	9
Social Workers	2
Region of Practice	
Central Canada	12
The Atlantic Provinces	5
The Prairie Provinces	10
The West Coast	3
Role in MAiD	
MAiD Assessors	2
MAiD Assessors and Providers	17
MAiD Coordinators	6
MAiD Team Members	5
Professional Background	
Palliative/Hospice Care	17
Family Medicine	6
Other	7

MAiD, Medical Assistance in Dying

### Data collection

After receiving ethics approval and informed consent, in-depth, semi-structured interviews of approximately one hour were conducted over Microsoft Teams or the telephone. They occurred between November 2020 and March 2021 prior to the proposed Bill C-7 legislation being enacted. An interview guide was used to explore participants’ thoughts and perceptions, including their values and ethical perspectives, about honouring eligible patients’ written agreement to receive MAiD following their loss of decision-making capacity. Specifically, participants were asked to share their experiences with eligible patients’ loss of capacity to consent, their thoughts on the proposed amendments that would waive the requirement of the final consent, and instances in which written agreements to waive the final consent with eligible patients could be appropriate or problematic. Short notes on contextual details were gathered following each interview. All interviews were audio recorded and transcribed verbatim.

### Data analysis

Data analysis began while conducting the interviews and during the process of validating the transcripts [29, 30]. A voice-centred relational approach, described by Mauthner and Doucet (1998) as a method that “keeps the respondents’ voices and perspectives alive while recognizing the researcher’s role in shaping the research process and product” (p. 119) [30], was used to analyse the transcripts. This approach consisted of two stages. During the first stage, four consecutive readings of the transcribed interviews were conducted; during each, the analysts ‘listened’ for different elements based on the concepts of power, relationality, and moral agency (p. 126) [30]. This process allowed a holistic analysis of each participant’s experiences that were captured in memos [30]. The second stage consisted of data summarisation and thematic analysis [30]. The themes generated following the readings focused on the individual experiences of participants and the researchers’ notes and memos. The process allowed the accommodation of similarities and variations in participants’ experiences of power, relationality, and expression of moral agency across the data [29, 30]. The themes were initially organized and recorded in NVivo™ for navigation. All methods were carried out in accordance with relevant guidelines and regulations.

### Findings

The participants (Table 5) included physicians (13), nurse practitioners (6), nurses (9) and social workers (2). Participant roles included assessors (2), assessors and providers (17), MAiD coordinators (6), and team members (5). The data analysis resulted in the following five main themes. See Table 6 for a summary and description of the themes.

**Table 6:** Themes and subthemes

Themes	Description
Balancing personal values and professional responsibilities	Participants supported expanding patients’ access to MAiD using the waiver of final consent. However, they anticipated ethical and legal challenges as well as discrepancies between public expectations and personal and professional limitations
Anticipating strengths and limitations of the proposed waiver of final consent amendment	Participants anticipated various benefits with the proposed amendments. Possible limitations as well as drawbacks of the proposed legislation were also discussed
Experiencing ethical influences on decisions to enter into written agreements with eligible patients	Decisions to enter into a written agreement with eligible patients to provide MAiD following their loss of capacity depended on factors such as familiarity with patients, interpretations of the foreseeability of death, opportunities for patients to change their minds and the scheduled date of death
Recognizing barriers to the enactment of MAiD in the absence of a contemporaneous consent	Participants who were willing to provide MAiD using written agreements indicated that their inability to establish intolerable suffering, objection from family members, and resistance from patients may prevent them from enacting MAiD
Navigating the potential for increased risks and burden	The challenges of participating in MAiD following the changes in legislation such as increased workload, risk for legal and professional liabilities and burnout came to light. Participants suggested increasing professional, legal and institutional support for healthcare providers

#### Balancing personal values and professional responsibilities

Improving access to MAiD for eligible patients who lose capacity aligned with the moral values of the participants. Declining MAiD to previously eligible patients who lost decision-making capacity was morally challenging and frustrating, especially when they were confident in their patients’ wishes, and when the family was wholly supportive. Having the option to waive the final consent was believed to increase opportunities for patients who were at risk of losing capacity to access MAiD. However, all participants anticipated professional, personal, or legal challenges with providing MAID in the absence of a patient’s final confirmation of consent immediately prior to provision. Some conveyed that Bill C-7 would continue to limit access to patients who suddenly experience changes in capacity prior to requesting MAiD and consequently were unable to enter into an agreement to waive final consent, as well as those who may lose capacity prior to meeting all of the eligibility criteria.

Participants indicated that the final consent requirement of Bill C-14 immediately prior to provision gave them comfort that MAiD requests and provisions were entirely patient-directed. Providers valued the opportunity for closure by sharing final words with their patients and for patients to share final words with their families and friends. Patients had the opportunity to participate in their own celebration of life, and to be in control of their life until the very end. Some referred to the process of requesting a final consent as a *“ceremony”* (P-9). In the absence of capacity, there were concerns that these elements that made MAiD deaths unique and meaningful would be lost. Some indicated that it would feel mechanical and *“more like [involuntary] euthanasia”* (P-6) or like *“turning off that ventilator”* (P-15) (i.e., withdrawing treatment). One provider shared, *“I think that's going to be really tough to do…kind of sneak up on someone when they are defenceless”* (P-8).

Participants believed that although the new legislation is well-intentioned, the challenges they may encounter have not been fully considered while establishing the criteria and safeguards. Providers wanted to be reassured that the written waiver of final consent arrangement they would enter into with their patients would provide legal protection. Some discussed strategies such as audio or video recorded confirmation of consent to shield themselves and their patients. Many, however, anticipated being powerless in doing what they believe is right for the patients when provisions are challenged legally by family members or other healthcare team members. As one provider highlighted, *“If one [challenge] went into battle with the [regulatory body] or the coroner's office or litigation, that would be the last MAiD case I would be involved with even if I was completely vindicated …there's going to have to be self-preservation”* (P-5).

Participants also discussed discrepancies between the expectations of the public and organizations that support MAiD and what healthcare providers are comfortable with, due to their moral values, as well as professional and legal limitations. One MAiD provider shared an encounter following a patient’s loss of capacity, and subsequent ineligibility for MAiD; *“In one of the letters that I received from the disgruntled group of friends, and this is I think quoted almost word-for-word, ‘how difficult could it be to find a physician in your hospital to provide three simple injections?’”* (P-3). Providers believed such expectations would increase with Bill C-7, thereby potentially restricting the moral agency of providers given societal pressures to provide.

#### Anticipating strengths and limitations of the proposed waiver of final consent amendment

With Bill C-14, patients reportedly endured distress and challenges due to their fear of losing capacity and being ineligible for MAiD. Patients often withheld or underused pharmacological therapy due to its impact on mental capacity, resulting in poor pain and symptom management. One participant shared, *“I think the way the legislation is set up right now [Bill C-14] …people have a fear of losing capacity and no longer having access to this option. That's part of their suffering, and so they pursue it earlier than they need to.”* (P-25). The written waiver of final consent agreement and the removal of the 10-day wait period were anticipated to enhance patients’ sense of control, relieve anxiety, and improve overall comfort while awaiting MAiD. The changes were also expected to potentially prolong lives by minimizing patients' need to access MAiD early in fear of losing capacity. Participants anticipated that patients would be empowered to attend special occasions or milestones without fear of becoming ineligible for MAiD due to the loss of capacity.

Participants shared that they endured guilt and frustration when socio-political elements such as professional or personal commitments, geographical constraints and organizational barriers resulted in their patients' capacity loss and subsequent ineligibility for MAiD. The waiver of final consent amendment was believed to widen their window of opportunity to provide MAiD. Participants also anticipated a reduction in the need to have uncomfortable conversations about expediting death due to the patients’ risk for capacity loss. Some revealed that they would enter into a written waiver of final consent agreement with all willing patients to avoid possible errors in prognosticating the likelihood of capacity loss and to account for other unforeseen situations. These participants believed that all patients who had a reasonably foreseeable death were at some degree of risk of losing capacity, and therefore would meet the criteria for waiver of final consent.

A few providers indicated that they may find less value in providing MAiD following patients' loss of capacity. These participants perceived that patients no longer suffer if they are in a state of unconsciousness. As one participant shared, *“To do it when somebody is unconscious because they're no longer suffering and so to do it for the family is something I would do, but I wouldn't feel like I had…had provided real value then.”* (P-17). Some believed that decisions to honour written agreements of incapacitated patients should depend on family preferences to support their grieving process. Others, however, advised that MAiD provisions should reflect a patient’s previously stated wishes rather than their family’s or healthcare providers’ values and beliefs. These participants highlighted the importance of ensuring that patients receive MAiD to prevent them from enduring a dying process that they had explicitly stated would be intolerable or *“abhorrent to them”* (P-5). To ensure that the provision occurred before their capacity loss, these providers indicated that they would continue to monitor for capacity loss and expedite MAiD.

Similarly, some providers expressed concerns that the proposed legislation would only allow provisions to occur on the date chosen by patients as indicated in the written agreement and that this could result in patients being left in a state of incapacity for an extended period of time, a condition many had hoped to avoid by choosing MAiD. To honour such requests, participants hoped to have the means to provide MAiD as soon as patients lose capacity (it was later clarified in Bill C-7 that this is possible). Participants who voiced such concerns either shared the same values as the patient or respected and understood their patients' beliefs that life was less meaningful once self-awareness or the ability to make relevant contributions was gone.

In addition, participants shared that with Bill C-14, family members suffered following patients' loss of capacity because of the need to hold vigil during their often-protracted death and the loss of opportunity to say their final goodbyes. Reportedly, some family members experienced guilt as they perceived they had failed to fulfil their family member’s wish to access MAiD and were burdened with making alternative end-of-life decisions on behalf of incapacitated patients. One participant shared, *“The patient’s wife just broke apart and she said to us, and we've heard this from other people as well, that ‘it's what he wants, and we've talked about it forever. I'm breaking my promise to him’ …it was heart wrenching to be in that position”* (P-6). These family members were believed to be at risk of experiencing complicated grief. Participants anticipated that the option of waiver of final consent would relieve family members of some burden and minimize the risk for complicated grief. Alternatively, participants who had experiences with family members who were opposed to MAiD, shared concerns that MAiD provisions following patients’ loss of capacity could complicate the grief that family members who preferred a natural death experience. MAiD coordinators and social workers shared that family members' needs, following MAiD provisions, were often neglected. In light of the changes in legislation, involving family members early on, identifying the substitute decision-makers, and informing them of the patients’ end-of-life wishes were perceived to be vital.

#### Experiencing ethical influences on decisions to enter into written agreements with eligible patients

Decisions to enter into written agreements with eligible patients depended on various ethical factors. An important influence was the eligibility assessment process. Longitudinal evaluations of patients' values and end-of-life wishes were believed to be required to honour written agreements for MAiD. One participant stated *“I think without [established relationships with patients and families] I would be a technician. I wouldn't be nearly as effective, and I would have moral distress about being a technician rather than a care provider for the patient and their family. It’s about the patient and their whole person, their family, their community, their spiritual selves, you know”* (P-22). Such assessments provided them the added benefits of a thorough understanding into the trajectory of the patients’ illness, the treatment options that were explored, and opportunities to establish trusting relationships. However, the feasibility of elaborate assessments often depended on patients' risk for capacity-loss or imminent death.

Some participants expressed concerns about the lack of rigour in the assessments conducted by some healthcare providers with Bill C-14. They worried that similar practices would be harmful to patients as the changes with Bill C-7 would require more robust assessments. The variations in the interpretation of foreseeability of natural death and grievous and irremediable suffering and how it may impact assessments, also concerned participants. They expressed discomfort in waiving the final consent for MAiD using a written agreement if they were unfamiliar with the patient. As one participant shared, *“I think I would have to be pretty knowledgeable of the person and their trajectory and the consistency. To just fly in and provide …I don’t think I would do that”* (P-3).

The inability of patients to change their mind due to the removal of the final consent requirement and the 10-day reflection period influenced a few participants’ decision to provide MAiD using written agreements to waive the final consent. Although most participants believed that patients who request MAiD and have set a date have given the decision due consideration, participants reported that there have been rare occasions when patients changed their minds. Additionally, some patients who are assessed to be eligible do not set a date or go through with MAiD. These patients were described as finding comfort in knowing they had the option if they needed it. Participants who had such experiences expressed caution for the need to be certain of the patient's wish prior to honouring written agreement to waive the final consent. They also emphasized that a peaceful end of life was possible even in the absence of MAiD.

The scheduled date of MAiD provision was anticipated to impact decisions to enter into a written waiver of final consent agreement. Most participants appreciated the requirement in Bill C-7 to establish a date for provision in the written agreement as they believed choosing a date on patients' behalf would be burdensome for the healthcare team and family. However, concerns about the lack of criteria and time limit for choosing a date were discussed. The close proximity of the scheduled date of provision to the eligibility assessment was anticipated to be reassuring to some practitioners. Alternatively, if the chosen date for MAiD was far in the future, participants expressed concerns that patients may not yet meet eligibility requirements such as a reasonably foreseeable death and intolerable suffering, or that patients’ wishes may change during a long wait period. Dates chosen around major life events or milestones were reported to be exceptions. Some believed that scheduled dates were arbitrary and that patients should be allowed to reschedule if they wish to do so. The importance of revisiting the written waiver of final consent agreement at regular intervals to assess for status changes and consistency in request until the scheduled date of provision was also highlighted.

#### Recognizing barriers to the enactment of MAiD in the absence of a contemporaneous consent

Various socio-political and legal constraints were anticipated to impact provision, once a decision to provide MAiD using the written waiver of final consent agreement was made. For many assessors, the ability to ascertain intolerable suffering influenced their eligibility decisions. Comfort levels in providing MAiD following patients’ loss of capacity depended on the providers’ perceptions of the patients’ quality of life and suffering. Therefore, participants anticipated challenges in providing MAiD to patients in a comatose state who appear to be pain and symptom-free or to patients in a conscious state of contentment following the loss of capacity, as in the case of dementia or other neurological conditions. One participant shared; *“I completely appreciate that people don’t want to become that other person [following capacity loss], but I don’t know how as a society we’re going to balance out the two sides of that. I think it’s going to add a lot of stress on assessors and providers”* (P-1). The need to decipher situations in which patients would want MAiD following their loss of capacity, rather than automatically providing MAiD to everyone who has a written agreement was discussed. Some, however, believed it was important to honour the patients' previously established wishes if they had explicitly stated that being in a state of unawareness would be intolerable to them, regardless of their perceived comfort level following loss of capacity.

The opposition of family members to MAiD provisions following the patients’ loss of capacity was also believed to impact patients’ access to MAiD. Such objections were believed to be similar to family members overriding advance care plans, often resulting in patients receiving treatments they had indicated they did not want. Although participants believed the right approach would be to honour patients’ wishes, providers shared that if family members objected persistently, they may not provide MAiD, due to the potential for legal or professional implications and the desire to avoid conflict. Additionally, participants had concerns about being unable to verify family members’ claims that patients had changed their mind before losing capacity, and no longer wanted MAID. Similarly, providers anticipated that family members or other healthcare team members may inaccurately perceive involuntary movements or actions by patients as resistance. One participant shared; “*All the family are saying, no, don't do this and there's no one in the bed that can look at you and say, yes, do this”* (P-28). Providers shared that their inability to fulfill their patients’ MAiD requests due to family members’ objections would be morally challenging for them.

Participants also expressed concerns about the mandate that requires practitioners to withhold the provision if patients resist in some meaningful way. Some believed that resistance or declining MAiD following the loss of decision-making capacity should be approached and assessed cautiously, as there is a possibility that the patient may have some degree of competence and may have changed their minds. However, challenges in discerning what actions or gestures indicate resistance were anticipated. Participants spoke of how confused patients may resist intravenous insertions or medications. Some participants believed these actions should not be interpreted as resisting MAiD. They pointed out that following the loss of capacity, patients who are anxious, delirious, or agitated, may be the ones that need the provision the most, as they are suffering. One participant shared *“these people who are combative and confused and stuff they're actually suffering the most, you know, requiring sedation on a regular basis. Those are the people who I think really need this”* (P-12). These participants suggested trialling symptom management strategies to comfort patients prior to proceeding with provision. Participants believed that variations in how healthcare providers perceive resistance would depend on their familiarity with the patient as well as their comfort with providing MAiD in the absence of a contemporaneous consent.

Participants were familiar with the logistical challenges as they had read about similar cases in the Netherlands. Most disclosed, citing their moral values and fear of legal repercussions, that they would not be able to physically or chemically restrain patients to provide MAiD even if it were legally permissible to do so. One participant suggested; *“I do not believe any healthcare provider can go in and if someone is resisting, hold their arm down and force an IV in. That is absolute moral distress. I think we need to be transparent with the patient and family up front to say the patient will not get MAiD because of [resistance]”* (P-18). However, concerns that the law does not specify if MAiD provisions should be attempted again at a different date or time if patients resisted were raised.

#### Navigating the potential for increased risks and burden

In the previous themes, the vulnerability of participants’ anticipated role, and the potential for ethical and legal risks and burdens following the introduction of Bill C-7 came to light. Those who were willing to provide MAiD using a written waiver of final consent agreement indicated that they would require support from their regulatory bodies, organizations and teams. Strategies mentioned included involving the ethics team and institutional lawyers to establish safeguards and policies to protect the team from repercussions. While some had access to such services, many did not. Participants anticipated that more frequent debriefing sessions would be required and that healthcare providers would need to take breaks from providing MAiD more often. MAiD coordinators and team members shared that they received support from Canadian Association of MAiD Assessors and Providers (CAMAP) and online support groups.

With the expanded access to MAiD, participants expected a drastic increase in workload and were concerned that this could further compromise the already understaffed and underfunded teams and result in burnout. The lack of adequate remuneration and support impact many practitioners under the current legislation. In Ontario, nurse practitioners reportedly receive no compensation for their MAiD work unless it is carried out as part of a salaried position. A MAiD coordinator shared the following concerns: *“We don't have physicians or NPs that do this as part of their job, they're just volunteering to be involved, usually on their weekends or evenings. And so, the thought of doing that and then being involved in a court case or something that's really distressing, I am really worried that people won't want to be involved and it's all voluntary. So, I'm trying to look at how are we going to assign cases to people in a way that's, ethical for the physicians and NPs but also doesn't limit access to patients in those scenarios”* (P-29).

Participants believed the transition from Bill C-14 to C-7 would require extensive resources to plan and the creation of policies and guidelines, which some indicated had not yet begun. They expressed concerns that too many changes were proposed at once, especially given the ongoing COVID-19 pandemic. Healthcare providers were reportedly not given proper training, adequate time to prepare or come to terms with the changes. Practices such as advance care planning, identification of substitute decision-makers, extensive assessment processes and follow-up care for families would be required with the changes in the legislation. While some teams already had these practices in place, others reported they needed to be established. Participants who were part of MAiD teams identified the benefits of using an interdisciplinary approach in offering counselling and support for patients and family members.

Participants expressed concerns that the increased professional responsibility and legal liability the changes would bring could deter healthcare providers from continuing to be a part of MAiD provisions. Non-provider team members believed the comfort level of providers in honouring MAiD using written agreements would vary depending on their values, beliefs and experiences, as one participant shared; “*my only concern in regards to advance directives [waiver of final consent] is that there will be a reduction in people who are willing to provide that and so it will be very much clinician dependent”* (P-19). Assessors and providers revealed that their willingness to enact written waiver of final consent agreement for MAiD would be based on a case-by-case approach. Some indicated they might stop participating in MAiD following the changes in legislation.

## Discussion

This study reveals that participants, in principle, supported waiver of final consent and discussed many perceived benefits. However, MAiD provisions, in the absence of direct patient input immediately prior to provision, were expected to change the experience of MAiD provision significantly. Participants foresaw various ethical challenges with its implementation and practice. This study highlights how healthcare providers’ moral agency, including their decisions to participate in MAiD using written waiver of final consent, is influenced by their personal values and experiences, power dynamics and relationality [25]. Similarly, previous studies have reported that socio-political, ethical and legal ramifications and lack of support influenced healthcare providers’ decisions to participate in MAiD [8, 32, 33].

Participants in this study discussed the vulnerability of eligible patients who were at risk of or fear losing capacity and hence, eligibility, under Bill C-14 given that it did not permit written agreements for MAiD [23]. Some participants believed patients’ ineligibility for MAiD following loss of capacity was a form of social injustice, as many of their patients had suffered while awaiting MAiD due to undertreated symptoms, while others chose to die earlier in fear of losing decision-making capacity, reflecting ethical concerns that also have been expressed in other reports [1, 34, 35]. The waiver of final consent amendment is anticipated to increase vulnerable patients’ power and control over their end of life and overall comfort during the end-of-life period [36]. However, patients are dependent on the healthcare provider’s willingness to waive final consent for MAiD [14, 23]. Liaschenko and Peter emphasize that power is morally neutral and can be legitimate if healthcare providers use their power to help patients meet their goals [25]. As in previous studies [19, 20, 37], most participants in this study were willing to honour written waiver of final consent agreements as it would support their patients’ right to access MAiD and aligned with their moral values and beliefs. However, participants shared concerns about various institutional constraints or legal safeguards, such as withholding MAiD if patients resist in a state of confusion, which may impede healthcare providers’ ability to provide patients with the kind of death they desired. Similar to the findings reported by Shaw and colleagues [37], some participants in this study indicated that the changes in the law should include access to MAiD to those who have lost decision-making capacity prior to requesting MAiD using advance directives.

Feminist ethics views persons as “interdependent, vulnerable and unequal in power” (p. 185) [25]. Vulnerabilities of older adults, especially those who have lost decision-making capacity, are often discussed in the literature [23, 36]. However, healthcare providers also experience vulnerabilities, especially while participating in contentious interventions such as MAiD. Participants felt helpless in declining MAiD to eligible patients following their loss of capacity with Bill C-14 and often received threats or demands to provide from family members [32, 37]. They experienced feelings of guilt in their inability to identify patients at risk for capacity loss and expediting the provision. Similarly, missing the opportunity to provide MAiD to patients who were in remote locations or in institutions where MAiD is not provided prior to their loss of capacity caused distress. The waiver of final consent amendment to the legislation is believed to increase healthcare providers' ability to offer patients the kind of death they desire and thereby, minimize distress healthcare providers experienced when MAiD could not be provided. The changes may also mitigate some of the anger and frustration of family members and patients often directed towards the healthcare providers when MAID was not possible.

However, the proposed amendments are believed to exponentially diminish healthcare providers’ moral agency due to a lack of resources, and the presence of legal and professional constraints. An increase in workload to address the potential legal and professional liabilities is predicted. The potential for burden is high due to unclear eligibility criteria and safeguards as well as socio-political constraints such as lack of resources, time, remuneration and support as has been noted in other studies [8, 13, 32, 38, 39]. In addition, patients and family members may have unrealistic expectations about access to MAiD and the role of the healthcare providers. Providers are often blamed when the provisions are not possible and are at risk for legal repercussions from objecting family members [8, 13, 32]. Assessors and providers require support from institutions, colleagues, regulatory bodies, patients, and families to provide MAiD using written waiver of final consent agreements [13]. As reported by Schuurmans and colleagues [13] in a Dutch study and by Frolic and colleagues [39] in the Canadian context, participants in this study revealed that they lacked the resources, time and support required to prepare for the enactment of the proposed legislation.

The findings from this study also point to the potential for vulnerabilities family members may experience. Providing MAiD to patients with capacity loss may further complicate the grief they experience [8, 40, 41]. Early, transparent communication with family members about patients’ wishes and follow-up visits with patients and their families can help minimize family members’ risk for complicated grief [8, 40]. However, such measures require time and resources that many lack [32, 38]. Patients and their families who were cared for by a multidisciplinary team, or those with palliative care backgrounds, had better support than others in previous studies [38, 42].

Numerous reports have highlighted the importance of assessing relational influences on autonomy while establishing patients’ eligibility for MAiD [1, 8, 32, 43]. The notion of relational autonomy also applies to healthcare providers who are making professional decisions [8, 25, 44]. Influences on decisions found in this study and others include familiarity with patients and a relationship of trust [8, 13, 19]. Due to the possibilities for changes in patients’ status and desire for MAiD, some participants expressed discomfort about the lack of clarity about the time limits on setting a date for provision in the written agreement. Similar concerns were reported by participants in a Dutch study [13]. Individual perspectives on criteria such as the foreseeability of death, the inability to determine intolerable suffering and the objective assessment of the quality of life following the loss of capacity have been found to impact decisions [8, 13, 15]. Providers in previous studies, have chosen to decline providing MAiD to patients who are perceived to experience some joy or ‘experiential pleasures’ following their loss of capacity, demonstrating how difficult it can be to predict someone’s future potential for quality of life [9, 11–15, 41, 45]. Most participants in this study indicated they would not honour written waiver of final consent agreements for similar reasons. Others anticipated challenges in providing MAiD to patients who appear comfortable following their loss of capacity or in a state of unconsciousness. Some providers, however, are willing to honour written agreements regardless of the level of perceived quality of life following the loss of capacity. These participants shared similar values as patients who explicitly indicated that they no longer wanted to live if they were unable to have ‘critical interests’ [45] such as achieving goals and dreams and living a meaningful life. Willingness to enter into written waiver of final consent agreements for MAiD also depended on the severity of risk for legal or professional repercussions that may occur if family members or others objected to provisions [12].

Our findings, similar to studies from the Netherlands [9, 13], suggest that the absence of patients’ capacity to consent at the time of provision may increase healthcare providers’ risk for moral distress. The waiver of final consent will change the experience of partaking in MAiD for many healthcare providers, as aspects of MAiD that they perceive to be morally acceptable will be altered. For instance, the requirement of contemporaneous consent with Bill C-14 reassured providers that provisions were entirely patient-directed [9, 41]. Healthcare providers' final interactions with patients and patients’ ability to say goodbye to their families prior to provision minimized the moral discomfort that some experienced. Similarly, healthcare providers valued the ability to ensure intolerable physical and existential suffering were met as legally required [9, 41]. Without patients’ input at the time of provision, healthcare providers will have the added responsibility of facing objections to MAiD provision from family members or other members of the healthcare team [9, 12, 32, 37]. Healthcare providers may also receive requests to partake in provisions that they may not be comfortable with [13, 46]. This study indicates that decisions to enter into and honour written agreements to waive final consent for MAiD would be made on a case-by-case basis, and patients’ access to MAiD would depend on the moral judgments of each assessor and provider. These findings highlight that while is important to expand the eligibility criteria, it is equally important to address and support the needs of healthcare providers to ensure equitable access to MAiD.

## Recommendations

This study highlights the importance of implementing strategies for the safety and wellbeing of healthcare providers to ensure equitable access to MAiD. MAiD team members should be provided adequate remuneration, time and resources to safely implement changes in the legislation and to support patients and family members [32, 37, 39, 42, 47]. Mandatory end-of-life care for patients and MAiD-related education are required for all healthcare providers to minimize the burden on palliative care and MAiD providers and improve end-of-life care [13, 37, 48]. Access to legal, professional, and institutional support may increase healthcare providers' willingness to participate [13, 39]. Introducing policies on early end-of-life conversations, advance care planning and identifying and documenting the correct substitute decision-makers would lessen family disagreements, delays in requests for MAiD and eligibility assessments [39]. MAiD team members should have access to psychological and emotional support and opportunities to debrief [13]. These strategies may, in turn, increase the sustainability of and access to MAiD and improve overall experiences with end-of-life for patients, family members, and healthcare providers.

## Limitations

The perspectives of healthcare providers were collected prior to the passing of Bill C-7. Their subsequent experiences with implementation of Bill C-7 may provide further or varied insight on the use of waiver of final consent in real life. Follow-up research is required to learn about the implementation and impact of Bill C-7 on the provision of MAiD and the experiences of patients, families, and healthcare providers. In addition, the perspectives of healthcare providers who had no experience with the provision of MAiD and those opposed to MAiD are not captured in this study.

## Conclusion

Numerous benefits of the proposed advance consent amendments have been identified in this study. Despite the growing concerns about the proposed expansion of MAiD [49], our findings reveal that healthcare providers are cautious in their approach to changes in the legislation. Participants anticipated the process of implementation to be complex. Some healthcare providers indicated that they may not continue to partake in MAiD or may provide on a case-by-case basis due to moral conflicts with the proposed changes, which could result in in decreased access to MAiD. The implementation of the changes in legislation may have occurred without the required training, support, and resources for many healthcare providers [39]. Further research is required to explore the impact of the Bill C-7 on healthcare providers. In addition, perspectives of healthcare providers need to be sought prior to the implementation and enactment of further changes to the MAiD legislation.

## Data Availability

The data that support this study are available from the corresponding author (CV) on reasonable request, subject to privacy and confidentiality commitments.

## Abbreviations

MAiD: Medical Assistance in Dying
P: Participant
NP: Nurse practitioner
